# Cardiovascular examination using hand-held cardiac ultrasound

**DOI:** 10.1007/s12574-021-00540-x

**Published:** 2021-08-02

**Authors:** Sam Jenkins, Mohamed G. Shiha, Eron Yones, James Wardley, Alisdair Ryding, Chris Sawh, Marcus Flather, Paul Morris, Andrew J. Swift, Vassilios S. Vassiliou, Pankaj Garg

**Affiliations:** 1 Department of Infection, Immunity and Cardiovascular Disease, University of Sheffield, Sheffield, UK; 2 Sheffield Teaching Hospitals NHS Foundation Trust, Sheffield, UK; 3 Norfolk and Norwich University Hospital, Norwich, UK; 4 Norwich Medical School, University of East Anglia, Norwich, UK; 5 Insigneo Institute for In Silico Medicine, Sheffield, UK

**Keywords:** Hand-held cardiac ultrasound, Left ventricular ejection fraction, Wall motion abnormality, Left ventricular hypertrophy, Valvular heart disease, Jugular venous pressure

## Abstract

Echocardiography is the first-line imaging modality for assessing cardiac function and morphology. The miniaturisation of ultrasound technology has led to the development of hand-held cardiac ultrasound (HCU) devices. The increasing sophistication of available HCU devices enables clinicians to more comprehensively examine patients at the bedside. HCU can augment clinical exam findings by offering a rapid screening assessment of cardiac dysfunction in both the Emergency Department and in cardiology clinics. Possible implications of implementing HCU into clinical practice are discussed in this review paper.

## Introduction

Historically, the cardiovascular history and examination were the primary methods for screening cardiovascular disease. The advent of hand-held cardiac ultrasound (HCU) enables augmentation of the cardiovascular assessment at the bedside. These HCU devices enable subjective and quantitative assessment of cardiac morphology and function with reasonable diagnostic accuracy, even when used by inexperienced operators with basic training [1–5].

Echocardiography remains the first-line imaging test for the assessment of suspected cardiac pathology. Standard transthoracic echocardiogram (TTE) allows the determination of cardiac dysfunction by assessing LV cavity size, wall thickness, valvular appearance, and function, as well as for the presence of abnormal blood flow within the heart [6]. HCU technology is rapidly advancing. These devices may ultimately bridge the gap between physical examination and TTE, allowing for more rapid clinical decision-making and a reduction in the rate of unnecessary TTE referrals.

In this comprehensive review, we aim to provide clinicians with a greater understanding of handheld ultrasound probe specifications and discuss the clinical utility of HCU for the augmentation of the physical cardiovascular examination. Devices available at the authors' institution were used to acquire images included in the current review.

## Available HCU hardware and specifications

**Fig. 1:**
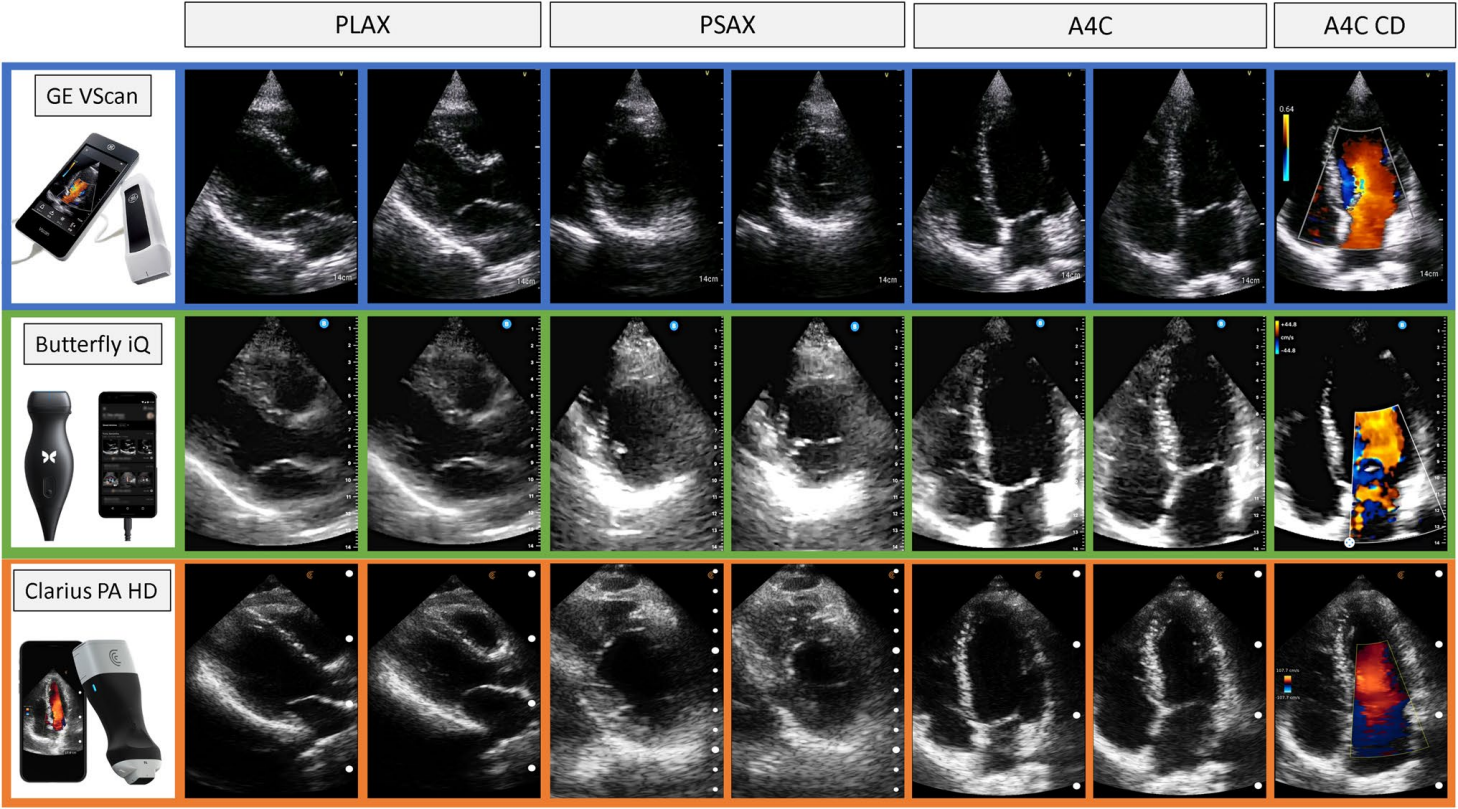
Comparison of main handheld echocardiographic systems available. *PLAX* parasternal long axis, *PSAX* parasternal short axis, *A4C* apical 4-chamber, *CD* colour Doppler

**Fig. 2:**
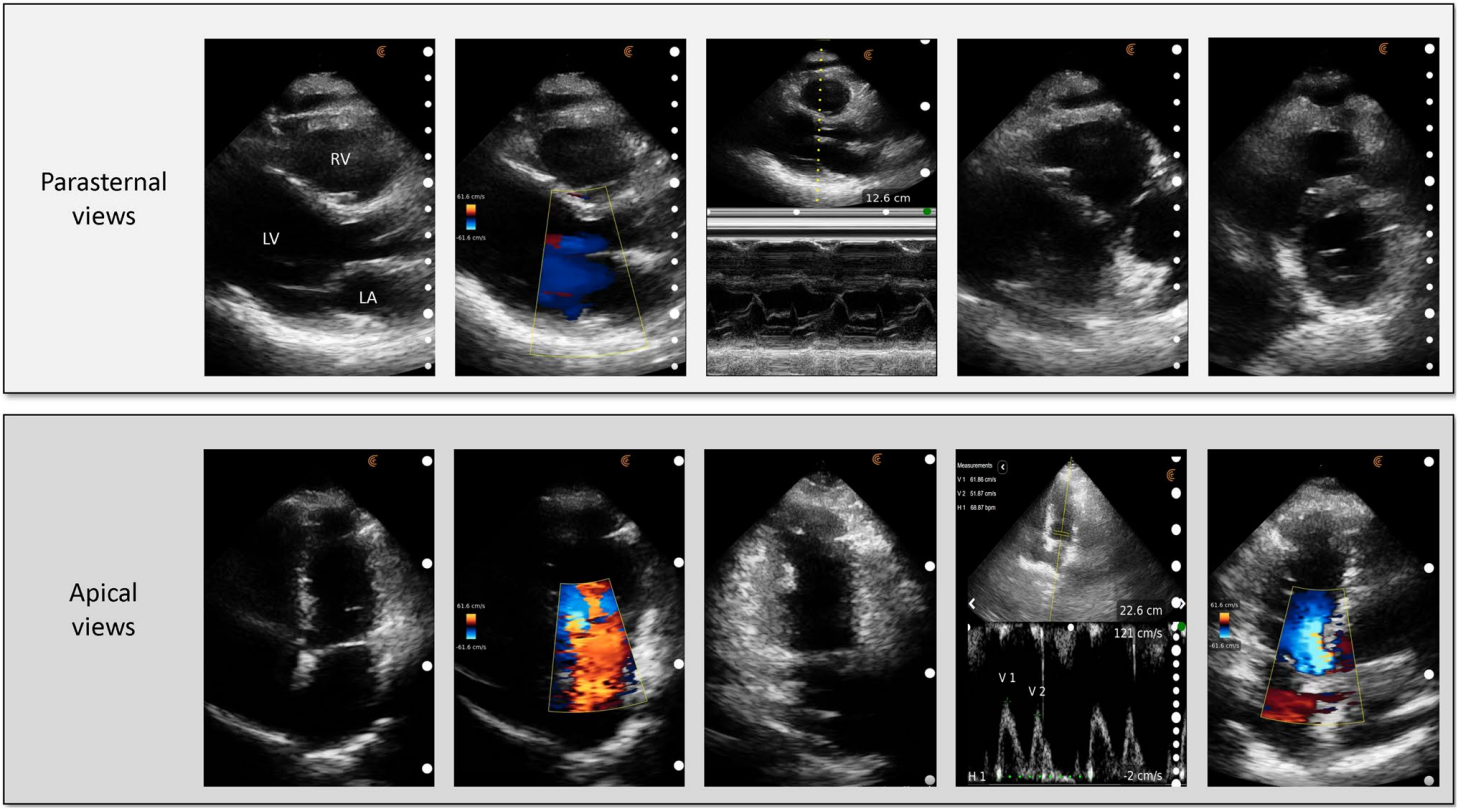
Comprehensive cardiac examination by Clarius PA HD. B-mode, colour Doppler and M-mode are demonstrated in parasternal views. In apical views, similar views and pulse wave Doppler for mitral inflow are demonstrated. V1 = peak early filling velocity, V2 = peak late filling velocity

Multiple HCU devices are currently approved for clinical use and widely available including Clarius wireless scanner (Clarius, Canada), Lumify (Philips, USA), Vscan extend (General Electric, USA) and Butterfly iQ (Butterfly Network, USA) (Fig. 1). The Lumify device is not available at our institution and therefore images from this HCU device have not been included. All these devices can acquire images in B-mode, M-mode and colour Doppler. However, each scanner has different capabilities and specifications (Tables 1, 2). A comprehensive set of images, including pulse-wave Doppler of mitral inflow performed using Clarius PA HD in a healthy individual is shown in Fig. 2.

**Table 1:** HCU hardware and specifications

Specifications	HCU device			
	Butterfly iQ	Philips Lumify	GE Vscan extend	Clarius C3 HD
Country	USA	USA	USA	Canada
Dimensions and weight	144 × 53 × 26 mm	C5-2 curved array (135 g)	Display unit (168 × 76 × 22 mm, weight 321 g)	164 × 78 × 38 mm
	313 g	L12-4 linear array (108 g)	Dual probe (129 × 39 × 28 mm, 120 g)	392 g
		S4-1 phased array (96 g)		
Price	~ £1699 + £360/year	~ $1200/6 − $ ~ 3360/24 months	~ €4250 − ~ €5100 + Tax	~ £4050
	1-year warranty	5 years warranty	1–3 years warranty	3 years warranty
Probe	Single probe (3 in 1)	3 probes (curved C5-2, linear L12-4, phased S4-1)	Dual probe (linear and sector)	Single probe
	CMUT/CMOS based			Waterproof
Cable	USB cable	USB cable	USB cable	Bluetooth and Wi-Fi
Compatibility	IOS, Android	Android	GE Vscan display	IOS, Android
Data storage	DICOM PACS	DICOM PACS	DICOM PACS	DICOM PACS
	Cloud storage	Cloud storage	Cloud storage	Cloud storage
Battery	120 min (2 continuous hours)		Continuous scan time of 60 min	Scan time: ~ 60 min
	5 h (for full recharge)		90 min and recharged in 75 min	Charge time: ~ 90 min
	Wireless charging		Exchangeable batteries for longer scan time	Standby: ~ 7 days

**Table 2:** HCU clinical imaging and artificial intelligence specifications

Specification	Functions	HCU device			
		Butterfly iQ	Philips Lumify	GE Vscan extend	Clarius C3 HD
Imaging	2D	√	√	√	√
	M-mode	√	√	√	√
	CDI	√	√	√	√
	PDI	√	x	x	√
	PWD	x	x	x	√
	Midline	√	x	x	√
	Measurements	√	x	√	√
	Scan depth (cm)	30	30	24	40
	Imaging presets (n)	20	10	15	8
Artificial Intelligence	Ejection fraction calculator	√	x	√	x
	Tele-guidance	√	√	x	√
	Auto optimise	x	√	√	√
	Bladder volume tool	√	x	√	x
	Apps from GE marketplace	x	x	√	x

*HCU* hand-held cardiac ultrasound, *2D* 2-dimensional, *CDI* colour Doppler imaging, *PDI* power Doppler imaging, *PWD* pulse-wave Doppler

Currently available HCU devices are designed to be portable and lightweight whilst maintaining high technical specifications. The Butterfly iQ device is manufactured using a complementary metal-oxide semiconductor (CMOS) material which allows mass production at a low cost, making this scanner one of the most affordable. HCU devices are compatible with Android, Android-like and iOS devices. Images can therefore be interpreted remotely by experienced diagnosticians. This includes utilisation in rural areas and low-resource countries where conventional ultrasound is not easily accessible [7, 8]. Devices also offer Tele-Guidance allowing live directions from a remote ultrasound expert when obtaining and interpreting images. The Clarius ultrasound scanner has the unique advantage of being wireless making the device more accessible. During the Coronavirus disease 2019 (COVID-19) pandemic, HCU has increased in popularity due to its safety as the size of the devices makes them easier to disinfect compared to standard echocardiography machines [9].

The ultrasound technology incorporated into HCU devices is becoming increasingly more sophisticated. GE Vscan and Butterfly iQ have developed technology to automatically calculate left ventricular ejection fraction whilst Clarius ultrasound scanner is the only HCU device able to provide pulsed-wave Doppler technology. Butterfly iQ have introduced an educational view guidance tool that assesses the quality of the images acquired in real-time to enhance scanning technique.

## Limitations of HCU devices

Battery life limits the use of HCU and therefore is indicated for quick scans. Vscan has an exchangeable battery which allows for longer scanning times compared to other devices. Continuous imaging can lead to the device freezing to allow for cooling which also limits the length of time they can be used.

Spectral Doppler technology causes HCU devices to heat up more rapidly and is one reason for only a few models incorporating these imaging modalities. Clarius is one of the only scanners on the market capable of PWD, permitted by the installation of a liquid heating device which prevents overheating [10]. This feature allows for quantitative assessment of cardiac pathologies including left ventricular filling pressures (i.e. diastolic function) calculated by measuring the E/A ratio and right ventricular dysfunction. This has the potential to offer significant value in the bedside assessment, diagnosis and monitoring of patients either with suspected or decompensating heart failure. Continuous-wave Doppler is also not a readily available feature of HCU devices. This modality would allow for quantitative assessment of high velocities across valves which could confirm aortic stenosis or regurgitation, mitral stenosis or tricuspid regurgitation. Currently, clinical suspicion of valvular heart disease or auscultation of murmurs can only be qualitatively assessed by the majority of HCU devices using standard B-mode and colour-flow Doppler.

The availability of HCU varies between departments, institutions and countries. For example, Butterfly is not commercially available in Japan compared to the UK where it occupies a large share of the market. Although this review aims to describe the range of specifications of individual HCU devices, there is little research into the impact of these varying specifications on their diagnostic ability.

## Clinical applications

Point-of-care ultrasound (POCUS) has rapidly transformed over the past two decades beyond the large machines that were initially introduced into emergency medicine and critical care. The advancement of POCUS technology has led to the introduction of more portable, handheld devices which are now also used in obstetrics to confirm intrauterine pregnancies, rheumatology to image for joint and soft tissue injuries and gastroenterology in the diagnosis of appendicitis and gallbladder pathology [11]. One of the most common indications for POCUS is suspected cardiac pathology. HCU is a focused, bedside, point of care examination which, in combination with clinical examination, ECG, blood results, and standard X-Ray can augment and enhance clinical diagnosis of a number of acute cardiac conditions. Positive and negative findings should be sought actively and documented clearly. Where there is doubt around HCU findings, these images should be reviewed by a senior sonographer or cardiologist. Intervention and treatment should not be commenced in cases where there is operator doubt about HCU imaging findings. With experience and increased caseload, HCU operator diagnostic and imaging skills will improve and over time, help provide good clinical data to allow early intervention and management of many conditions, aid discharge without need to wait for formal departmental TTE as well as help rapidly rule out various cardiac conditions.

### LV size and systolic function

Physical signs of impaired LV systolic function often only manifest when there is advanced disease. HCU can identify evidence of declining cardiac function in asymptomatic patients allowing for earlier commencement of treatment, thus limiting the development of cardiac failure. HCU permits the assessment of left ventricular morphology and function by estimating LVEF and detecting regional wall motion abnormalities (WMA) [4, 12–14]. These methods can yield greater clinical value compared to physical examination [15]. Auscultation of S3 is rarely found in patients with LV systolic dysfunction whilst pulmonary oedema is an insensitive sign and may only present when the disease is severe [16].

Rapid HCU assessment and identification of regional WMAs may help confirm a diagnosis of acute myocardial infarction (MI) in patients with chest pain who have an equivocal or non-localising ECG and/or indeterminate laboratory markers [17]. HCU may also offer initial estimates of baseline LV function following MI [18]. Assessment of LV function and presence of LV thrombus after myocardial infarction, especially anterior ST-segment elevation myocardial infarction, is important for both treatment and prognostic considerations. Detection of poor LV systolic function favours a diagnosis of heart failure (HF) instead of pneumonia or chronic obstructive disease exacerbation in patients presenting with non-specific symptoms. This subgroup of patients could subsequently benefit from earlier referral for further imaging and potentially avoid unnecessary tests. The impact of valvular disease on cardiac function can be quantified by estimating LVEF. Periodic repeat assessment using HCU may be able to give an indication of advancing disease severity. These assessments can be conducted by specialist nurses in outpatient clinics or in the community providing potential detection of worsening function and referral to TTE before the patient becomes symptomatic [19–21]. Concurrent assessment of left ventricular hypertrophy (LVH) and LV dilatation when evaluating LV function can help grade the severity of cardiac disease or confirm clinical suspicions when individual findings are ambiguous.

### LV diastolic assessment

Routine TTE examination for LV filling pressure involves tissue Doppler imaging (TDI) to measure LV myocardial velocities (eʹ and aʹ), pulse-wave Doppler for mitral inflow velocities (E and A) and continuous-wave Doppler for measuring peak TR velocity. Current probes do not offer all these ultrasound technologies, hence LV diastolic assessment is limited to mitral inflow assessment by some probes which offer pulse-wave Doppler assessment. Nevertheless, left atrial size can be estimated by HCU and this is also routinely advocated for estimating LV filling pressures.

### Left atrial assessment

Left atrial assessment can be made easily by apical 4-chamber and 2-chamber views. These views are relatively simple to acquire by HCU. These views allow us to quantify both the left atrial area and volume. HCU can more sensitively detect left atrial enlargement compared with auscultation of S4 on clinical examination [14, 22–25]. Grading severity of mitral valve disease can be aided by measuring the diameter of the left atrium. Repeat imaging can assess the response to intervention post-surgery.

### Valvular heart disease assessment

Quantitative assessment of valvular heart disease (VHD) using HCU is limited because pulse-wave and continuous-wave Doppler modalities are not available with the majority of probes. Imaging for valvular insufficiencies or morphological abnormalities of stenotic valves, therefore, relies on qualitative colour-Doppler and B-mode assessment. Initial assessment following a clinical suspicion of VHD can determine whether the patient requires further investigation. Repeat imaging can also provide complementary information to clinical examination regarding disease progression, which can support a decision to refer for more complex imaging.

### Right ventricular size and function

Early detection of right ventricular (RV) enlargement and hypokinesis can improve the prognosis of acutely unwell patients such as those admitted with an undiagnosed pulmonary embolism (PE). The majority of fatal PEs occur within the first few hours of developing symptoms, with 30–50% remaining undiagnosed until autopsy [26, 27]. Rapid and accurate assessment of the RV using HCU can lead to a more efficient diagnosis, administration of treatment and improved prognosis, as findings can necessitate the urgent results of tests such as D-dimer in high-risk patients. RV end-diastolic diameter has been shown to be the most sensitive predictor of PE which can be measured using HCU. Subjective measures including hypokinesis and McConnell’s sign of free wall dyskinesia show poor sensitivity for the diagnosis of PE. Tricuspid annular plane systolic excursion shows reasonable sensitivity and could be measured using the M-mode modality of HCU devices. TTE does allow for quantitative assessment of RV dysfunction by measuring the tricuspid regurgitant jet velocity. This is almost exclusive to TTE as it requires continuous-wave Doppler [28, 29]. Estimates of systolic pulmonary arterial pressure (sPAP) to detect pulmonary arterial hypertension (PAH) have shown only moderate diagnostic accuracy when performed using HCU devices capable of CWD [18, 30, 31]. Low thresholds for bedside HCU assessment of patients with non-specific signs and symptoms may allow for earlier TTE referral and diagnosis of PAH, which is commonly only diagnosed at an advanced stage [32].

### Pericardial disease

The presence of an anechoic space within the pericardium, right atrial inversion or right ventricular diastolic collapse indicates pericardial effusion. These measurements using HCU can specifically predict pericardial effusions [14, 18, 33]. One limitation includes epicardial fat being misinterpreted as an effusion, especially when the operator is less experienced. Assessing respiratory variation of the mitral or tricuspid inflow velocities and the aortic flow velocity is limited to pulse-wave Doppler probes. Few HCU devices can offer this function whilst TTE routinely examines these entities allowing for quantitative assessment of pericardial disease. Assessment for pericardial fluid or tamponade in patients with myocardial infarction, myocarditis and pericarditis are important to guide investigation, therapy and discharge planning.

### Haemodynamic assessment

**Fig. 3:**
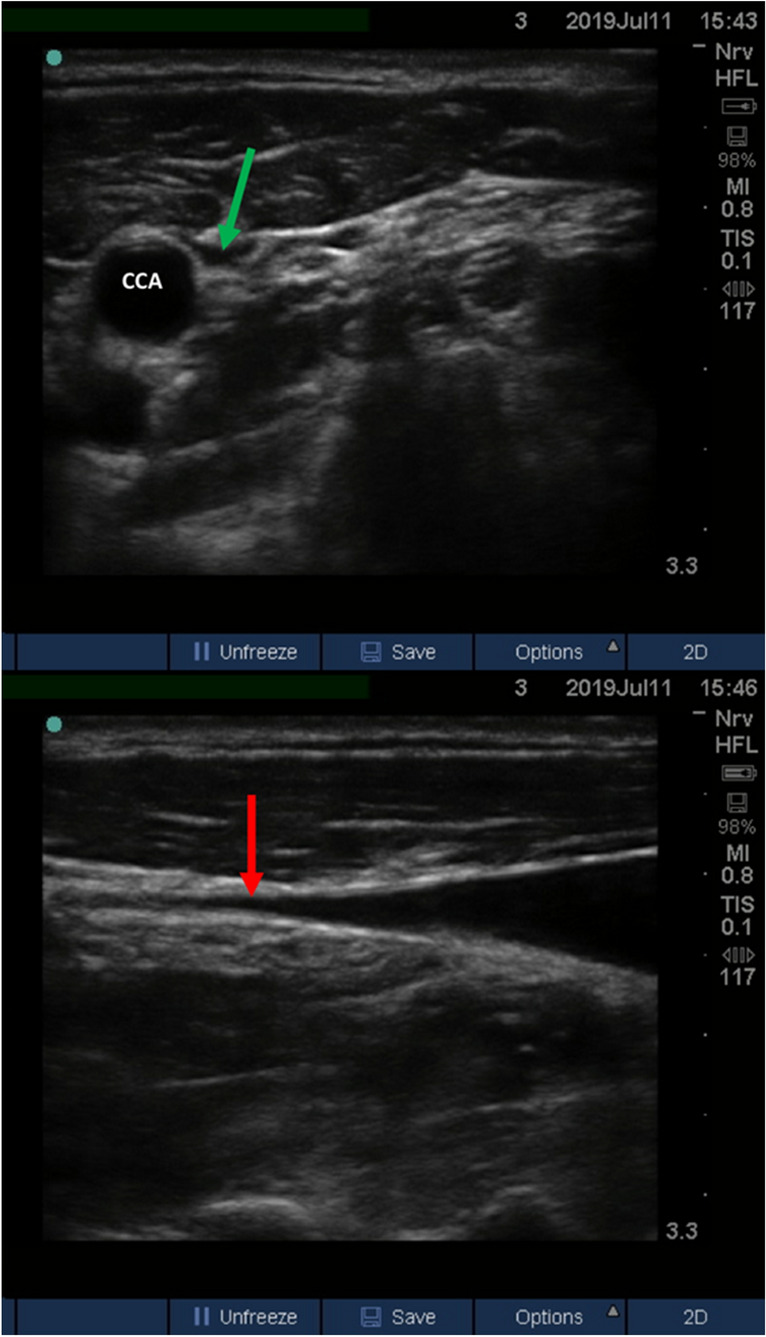
Ultrasound-measured jugular venous pressure imaged in a semi-recumbent position. Top: transverse view of a collapsed IJV. The green arrow points towards the collapsed IJV. Bottom: longitudinal axis of the IJV. The IJV tapers until reaching the point of collapse. The red arrow indicates the point at which the vein is collapsed. *CCA* common carotid artery, *IJV* internal jugular vein

Ultrasound examination of jugular venous pressure (U-JVP) can be used to estimate a patient’s fluid status by identifying the collapse point of the internal jugular vein (IJV). The collapse point is viewed in the longitudinal axis with the patient positioned in a semi-upright position (Fig. 3). The location of the jugular venous pulsations is highly subjective and accuracy of the physical examination varies significantly between clinicians [34]. HCU removes this subjectivity and can be easily viewed even by inexperienced operators. U-JVP allows visualisation of the venous collapse which would otherwise be undetectable by a physical examination in obese patients. However, U-JVP has limitations as estimates of fluid status become less specific in hypovolaemic patients [35]. In an emergency setting, the patient may be required to lie supine. In this scenario, U-JVP would not be viable and imaging of inferior vena cava (IVC) diameter and collapsibility would be required to estimate fluid status. IVC assessment in the subcostal view is the standard non-invasive bedside imaging method for estimating haemodynamic status. A greater amount of training to achieve basic competence in IVC image acquisition may be required compared to simple imaging of the jugular venous collapse point. However, IVC diameter and collapsibility can give a quantitative measure of fluid status and once sufficiently trained, sensitivity of the technique can reach up to 93% by experienced cardiologists [13]. In contrast, the accuracy of the height of the collapsing jugular vein above the right atrium as a measure of fluid status has been variably reported and may best be used as a simple, quick-look, qualitative assessment [35, 36].

## Training

Minimal training is required to gain basic competency in image acquisition using HCU [33]. Although 20–30 scans have been deemed acceptable to develop basic skills in ultrasound, accurate and reliable interpretation of images requires repeated exposure over a prolonged period of time [37]. The ability of images to be stored enables less experienced operators to request interpretation by more senior colleagues. This can limit the number of false-positive results which commonly occur among inexperienced users, especially when only having undertaken limited training [38]. Experience has been repeatedly shown to impact the diagnostic accuracy of HCU examinations. Level II/III echocardiographers are able to more accurately diagnose cardiac pathology compared to inexperienced operators with limited echocardiography training [33, 39–41]. This suggests that standard echocardiography training is fundamental to competently acquire and interpret images, as opposed to training solely with an HCU device. Positive results need to be interpreted with caution, especially when performed by inexperienced HCU users. Clinical decision-making should not be solely guided by the interpretation of HCU images, even by experienced echocardiographers. Instead, HCU should act as an initial diagnostic test to aid decision-making on whether further investigation including TTE and treatment is required.

The lack of a designated HCU training pathway, certification and hospital service poses a barrier to the implementation of bedside imaging in clinical practice. Levels of competence cannot be standardised and the absence of HCU services means clinicians cannot gain the experience necessary to acquire and interpret images with consistently high accuracy. Ultrasound education has been implemented in American medical school curriculums for more than a decade which is currently not available in the UK [42]. Such training aids students’ understanding of clinical anatomy and development of basic ultrasound skills as well as their diagnostic ability [5, 43]. However, this relies on sufficiently trained tutors being available which may incur geographical bias between schools. HCU services must also be available upon graduation to maintain their level of skill.

## Cost-effectiveness

HCU has been associated with reduced costs when compared with standard echocardiography [15, 44, 45]. Verification of clinical exam findings using HCU means that the rate of unnecessary TTE referrals could be reduced [15]. It is important to stress that HCU does not replace TTE and any abnormal or uncertain findings should be confirmed with further imaging. An increase in the use of HCU imaging could lead to a paradoxical increase in TTE referrals due to a rise in the number of incidental findings. The use of HCU by community nurses may enable further savings by monitoring patients with HF. This could allow for earlier detection of advancing disease, reducing the risk of further complications and need for more complex interventions. A substantial potential cost relates to the implementation of HCU services and provision of the appropriate training. Restructuring of the standard echocardiography pathway and development of standardised levels of competency are required to integrate HCU into routine practice, which has complex logistical and financial implications.

## Conclusion

The increasing sophistication of available HCU devices enables clinicians to more comprehensively examine patients at the bedside. HCU can augment clinical exam findings by offering a rapid screening assessment of cardiac dysfunction in both the Emergency Department and in cardiology clinics. Possible implications of implementing HCU into clinical practice are earlier detection of cardiac pathology and a reduction in unnecessary TTE referrals.
